# Can machine learning models improve the prediction of surgical site infection in abdominal surgery than traditional statistical models?

**DOI:** 10.1177/03000605241293696

**Published:** 2024-11-17

**Authors:** Pongsathorn Piebpien, Amarit Tansawet, Oraluck Pattanaprateep, Anuchate Pattanateepapon, Chumpon Wilasrusmee, Gareth J. Mckay, John Attia, Ammarin Thakkinstian

**Affiliations:** 1 Department of Clinical Epidemiology and Biostatistics, Faculty of Medicine Ramathibodi Hospital, Mahidol University, Bangkok, Thailand; 2 Department of Research and Medical Innovation, Faculty of Medicine Vajira Hospital, 292577 Navamindradhiraj University, Bangkok, Thailand; 3 Department of Surgery, Faculty of Medicine Ramathibodi Hospital, Mahidol University, Bangkok, Thailand; 4 Centre for Public Health, School of Medicine, Dentistry and Biomedical Sciences, Queen’s University Belfast, Belfast, UK; 5 School of Medicine and Public Health, and Hunter Medical Research Institute, University of Newcastle, New Lambton, New South Wales, Australia

**Keywords:** Surgical site infection, abdominal surgery, prediction model, logistic regression, machine learning, feature importance

## Abstract

**Objective:**

To externally validate by revision and update the study on the efficacy of nosocomial infection control (SENIC) model of surgical site infection (SSI) using logistic regression (LR) and machine learning (ML) approaches.

**Methods:**

A retrospective analysis of hospital database-derived data from patients that had undergone gastrointestinal, colorectal and hernia surgeries (identified by ICD-9-CM). The SENIC index was calculated and fitted in an LR. MLs were developed using decision-tree (DT), random forest (RF), extreme-gradient-boosting (XGBoost) and Naïve Bayes (NB).

**Results:**

The prevalence of an SSI was 3.21% (404 of 12 596 surgeries; 95% confidence interval [CI] 2.91%, 3.53%). The C-statistic for the original SENIC model was 0.668 (95% CI 0.648, 0.688) with an observed/expected (O/E) ratio of 0.998 (interquartile range [IQR] 0.750, 1.047). An updated-SENIC-LR model with six predictors had a C-statistic of 0.768 (95% CI 0.745, 0.790) and O/E ratio of 0.999 (IQR 0.976, 1.004). The performance of MLs considering 14 predictors was poorer than the updated-SENIC-LR with C-statistics of 0.679, 0.675, 0.656 and 0.651 for NB, XGBoost, RF and DT, respectively. Overfitting was detected for ML approaches, particularly for DT, RF and XGBoost.

**Conclusion:**

The updated-SENIC-LR model and NB may be useful for monitoring SSI risk following abdominal surgery.

## Introduction

Surgical site infection (SSI) may occur post-surgery,^1^ which is associated with an increased mortality risk of between 1% and 10% depending on the surgical procedure and wound classification.^2–9^ Previous reports indicate that SSI occurs more commonly following abdominal surgery, varying between 5.3% and 25.4%.^10–13^

Multiple approaches are used clinically to reduce SSI including antibiotic prophylaxis, modification of surgical technique and peri-operative optimization. A surgical risk calculator (SRC) can be used to identify patients at the highest SSI risk to aid targeted mitigation strategies. A scoping review of SSI prediction models (see supplementary materials, Figure S1) was undertaken and it identified 33 potentially relevant studies with 87 predictive models, 49 of which were previously externally validated. For SSI risk prediction following abdominal surgery, the national nosocomial infections surveillance (NNIS) and the study on the efficacy of nosocomial infection control (SENIC) models, derived from logistic regression (LR) approaches, provided the best discriminative performance following external validation with C-statistics of 0.707 and 0.683, respectively.^14^ Both models included similar predictors but the SENIC model was more easily applied given the predictors included were more commonly available following routine surgery with operation time classified as >2 h instead of actual measured time in the NNIS model. We wanted to test the accuracy of the SENIC model in our local setting, in addition to externally validating model performance and consistency.

Recent machine learning (ML) approaches have been used for improving risk predictive performance relative to traditional LR approaches.^15–17^ ML may help address some of the limitations associated with traditional LR including the assessment of non-linear associations between predictors and outcomes, multi-collinearity among predictors or residual effect-modifiers. However, there has been variability in the performance measures between ML and LR approaches.^15–18^

Therefore, this current study was conducted with the following aims: (i) to externally validate the SENIC model; (ii) to update the SENIC model with re-estimated coefficients and additional predictors derived from the LR and ML approaches; (iii) to compare the performance of SSI risk prediction between LR and ML models to identify the best performing model with potential clinical utility.

## Patients and methods

### Study design and data sources

This retrospective cohort study retrieved abdominal surgical data from the information system of Ramathibodi Hospital, Mahidol University, Bangkok, Thailand between October 2013 and December 2019. The inclusion criteria were as follows: (i) patients were aged > 18 years; (ii) patients had received primary abdominal surgery with contaminated wounds (i.e. gastrointestinal, colorectal surgery) and/or they had a cleaned-wound with a foreign body (i.e. hernia repair); (iii) patients were identified from the hospital databases using ICD-9-CM (see supplementary materials, Table S1).

Previous SRCs were identified from a scoping review (see supplementary materials, Figure S1) and the SENIC model was selected for external validation as follows:

$$ln\left[\frac{P}{1-P}\right]=-4.48+1.12x\mathrm{Abdominal}+1.04x\mathrm{ORTime}+1.04x\mathrm{WoundClass}+0.86x\mathrm{Ndiagnosis}$$

where Abdominal = abdominal surgery versus non-abdominal surgery, ORTime =operation time > 2 versus  ≤ 2 h, WoundClass = wound class contamination/dirty versus clean/clean-contamination, and Ndiagnosis = number of diagnoses ≥3 versus <3.

Predictive factors were retrieved from hospital and operation databases, which were categorized into three domains as follows: demographic data, pre-surgical data and peri-/post-surgical data (see supplementary materials, Table S2). Demographic data consisted of age and sex. Pre-surgical data consisted of risk behaviours, underlying diseases, emergency status, American Society of Anesthesiologists (ASA) classification and medication used. Peri-/post-surgical data consisted of ICD-9-CM for procedures, wound class, anaesthesia type, operation time, blood loss and transfusion, complications during surgery and admission ward after surgery. The outcome of interest was SSI within 30 days after surgery, which was identified from two sources as follows: (i) infection control nurse’s record; and (ii) electronic hospital databases. Patients’ discharge summaries and follow-up data along with diagnosis (ICD10), procedure ICD9 (e.g. open wound, dressing), antibiotics used and laboratory findings were employed to identify and verify SSI.

This study was reported in accordance with the transparent reporting of a multivariable prediction model for individual prognosis or diagnosis (TRIPOD) checklist.^19^ The study received ethical approval from the Faculty Ethics Committee of Ramathibodi Hospital, Mahidol University, Bangkok, Thailand prior to commencement (no: MURA2019/1087; no. MURA2024/616). Informed consent was waived by the Faculty Ethics Committee due the retrospective nature of the study.

### Data management

Data were retrieved and exported to My SQL 8.0 to process data encryption, data linkages, factor and outcome extraction. Missing data were imputed assuming missing at random using a multiple imputation with chain equation. Statistical models were constructed according to type of missing data including logit, multi-logit and linear regressions.^20^

### Statistical analyses

All statistical analyses were performed using the STATA statistical software, version 16.1 (StataCorp LLC., College Station, TX, USA) for Windows®. Data are summarized as mean ± SD or median (interquartile range [IQR]) for continuous variables as appropriate and frequency (%) for categorical variables. Continuous data were compared using Student’s *t*-test. Categorical data were compared using χ^2^-test. A *P*-value <0.05 was considered statistically significant.

External validation was performed as follows: (i) the SENIC score was calculated and fitted against SSI; (ii) and then the score was revised by re-tuning the model coefficients against the data, with revision following the inclusion of additional factors to the models including blood transfusion, diabetes mellitus, concurrent procedures, emergency and open surgery.^21,22^ Backward elimination was used to retain factors and generate a parsimonious model.

Model performance such as calibration and discrimination were estimated for each step.^21,22^ C-statistics measured the level of discrimination between SSI and non-SSI patients; the observed/expected (O/E) ratio measured calibration or closeness between the predicted- and the observed-values.^23^

Machine learning approaches were developed to estimate SSI risk that included models based on decision tree, random forest, extreme-gradient-boosting (XGBoost) and Naïve Bayes. Data were randomly divided into training and testing datasets according to a 70%:30% split, respectively. To preserve the natural distribution of risk factors and SSI events, up-sampling and down-sampling techniques, which would artificially alter the case-control ratio, were not employed. Recognizing the low incidence rate of SSI (approximately 3%), a ‘balanced' class weight parameter was used during model training to minimize bias towards the majority class (patients without SSIs).

The models derived from the training dataset were further refined through hyperparameter tuning. Grid Search Cross-Validation using the scikit-learn library was employed to systematically explore the hyperparameter space. Following this, a manual search was performed among the top-ranked candidates identified from the Grid Search Cross-Validation to optimize model performance and minimize overfitting on the testing dataset. This optimization process involved careful evaluation of all relevant metrics.

Model evaluation was performed by estimating recall (i.e. sensitivity), specificity, precision (i.e. positive predictive value), F1 scores and C-statistics using the testing dataset. Feature or factor contribution was evaluated using SHapley Additive exPlanations (SHAP) values.^24,25^ ML model performance was compared with traditional LR approaches (i.e. updated-SENIC-LR model). All analyses were performed by Anaconda 2020.07 for 64-bit Windows with Python 3.7 (Anaconda Inc., Austin, TX, USA).

## Results

A total of 42 246 abdominal surgeries were initially retrieved consisting of 18 795, 9884, 8061 and 5506 gastrointestinal surgeries, colorectal surgeries, hernia repairs and appendectomies, respectively. Applying eligibility criteria resulted in a total of 12 596 surgeries (10 704 patients) included in this current analysis. The demographic and clinical characteristics of the patients are presented in Table 1. There was a higher proportion of males (7048 of 12 596 surgeries; 55.95%) and the cohort had a mean ± SD age of 58.94 ± 15.59 years. The majority of patients that underwent surgery were designated ASA class 3 or lower (11 548 of 12 596 surgeries; 91.68%) and had concurrent procedures < 2 (8464 of 12 596 surgeries; 67.20%). Only 504 of 12 596 (4.00%) surgeries had diabetes mellitus. The majority of operative procedures were elective (10 693 of 12 596 surgeries; 84.89%) and undertaken using an open-surgical approach (11 111 of 12 596 surgeries; 88.21%). Approximately one-third (4798 of 12 596 surgeries; 38.09%) received antibiotic prophylaxis. Almost half of the operation times were ≤2 h, most had clean-contaminated wounds, general anaesthesia and were admitted to non-ICU wards postoperatively. Only 10.84% (1365 of 12 596 surgeries) required blood transfusions.

**Table 1.** Demographic and clinical characteristics of patients used to externally validate the study on the efficacy of nosocomial infection control (SENIC) model.

Characteristic	Total cohort *n* = 12 596	SSI group *n* = 404	No SSI group *n* = 12 192	Statistical analyses^a^
Sex				
Female	5548 (44.05)	200 (3.60)	5348 (96.40)	*P* = 0.025
Male	7048 (55.95)	204 (2.89)	6844 (97.11)	
Age, years	58.94 ± 15.59	59.77 ± 14.28	58.91 ± 15.63	NS
ASA class				
1	2037 (16.17)	31 (1.52)	2006 (98.48)	*P* < 0.001
2	4588 (36.42)	145 (3.16)	4443 (96.84)	
3	4923 (39.08)	178 (3.62)	4745 (96.38)	
4	997 (7.92)	45 (4.51)	952 (95.49)	
5	51 (0.40)	5 (9.80)	46 (90.20)	
Diabetes mellitus				
No diabetes mellitus	12 092 (96.00)	369 (3.05)	11 723 (96.95)	*P* < 0.001
Diabetes mellitus	504 (4.00)	35 (6.94)	469 (93.06)	
Number of diagnoses				
<3	12 355 (98.09)	386 (3.12)	11 969 (96.88)	*P* < 0.001
≥3	241 (1.91)	18 (7.47)	223 (95.53)	
Emergency				
Elective	10 693 (84.89)	287 (2.68)	10 406 (97.32)	*P* < 0.001
Emergency	1903 (15.11)	117 (6.15)	1786 (93.85)	
Pre-operative antibiotics				
No antibiotics	7798 (61.91)	221 (2.83)	7577 (97.17)	*P* = 0.002
Antibiotics	4798 (38.09)	183 (3.81)	4615 (96.19)	
Surgical approach				
Open surgery	11 111 (88.21)	332 (2.99)	10 779 (97.01)	*P* < 0.001
Non-open surgery	1485 (11.79)	72 (4.85)	1413 (95.15)	
Concurrent procedures				
<2	8464 (67.20)	177 (2.09)	8287 (97.91)	*P* < 0.001
≥2	4132 (32.80)	227 (5.49)	3905 (94.51)	
Operation time, h				
≤2	7044 (55.92)	73 (1.04)	6971 (98.96)	*P* < 0.001
>2	5552 (44.08)	331 (5.96)	5221 (94.04)	
Wound class				
Clean/clean-contaminated	11 112 (88.22)	372 (3.35)	10 740 (96.65)	*P* = 0.014
Contaminated/dirty	1484 (11.78)	32 (2.16)	1452 (97.84)	
Epidural anaesthesia				
General anaesthesia	12 542 (99.57)	404 (3.22)	12 138 (96.78)	NS
Epidural anaesthesia	54 (0.43)	0 (0.00)	54 (100.00)	
ICU				
Non-ICU	11 409 (90.58)	334 (2.93)	11 075 (97.07)	*P* < 0.001
ICU	1187 (9.42)	70 (5.90)	1117 (94.10)	
Blood transfusion				
No blood transfusion	11 231 (89.16)	259 (2.31)	10 972 (97.69)	*P* < 0.001
Received blood transfusion	1365 (10.84)	145 (10.62)	1220 (89.38)	

Data presented as mean ± SD or *n* of surgeries (%).

a Continuous data were compared using Student’s *t*-test; categorical data were compared using χ^2^-test; NS, no significant between-group difference (*P* ≥ 0.05).

SSI, surgical site infection; ASA, American Society of Anesthesiologists; ICU, intensive care unit.

The prevalence of SSI was 3.21% (404 of 12 596 surgeries; 95% confidence interval [CI]: 2.91%, 3.53%). The distribution of risk factors and SSI were investigated and demonstrated a significant association was observed between ASA class and SSI risk (*P* < 0.001) (Table 1). Patients with higher ASA class 3–5 experienced significantly higher SSI rates when compared with those patients with lower ASA class 1–2 (SSI rates of 3.62–9.80% versus 1.52–3.16%, respectively) (*P* < 0.001). Patients with diabetes mellitus had a significantly higher SSI risk than patients without diabetes mellitus (6.94% versus 3.05%, respectively) (*P* < 0.001). Patients with ≥3 diagnosed conditions had a significantly higher SSI rate than patients with < 3 diagnoses (7.47% versus 3.12%, respectively) (*P* < 0.001). Emergency cases and the number of concurrent procedures of ≥2 had significantly higher SSI risks than non-emergency cases and those with <2 concurrent procedures (6.15% versus 2.68%; 5.49% versus 2.09%; respectively) (*P* < 0.001 for both comparisons). SSI rates were a significantly higher in clean/clean-contaminated and in those receiving antibiotic prophylaxis than contaminated/dirty wounds and non-antibiotic prophylaxis with rates of 3.35% versus 2.16% (*P* = 0.014) and 3.81% versus 2.83% (*P* = 0.002). ICU admission, operation time >2 h and blood transfusion were all significantly associated with a 2–6 fold increased risk of SSI.

External validation of the SENIC model was performed as follows (Table 2): first, the original SENIC score (called model M_0_) was fitted against SSI providing a C-statistic of 0.668 (95% CI 0.648, 0.688) and O/E ratio of 0.998 (IQR 0.750, 1.047). Secondly, the SENIC model was revised by the addition of two of the four original predictive factors significantly associated with SSI (called M_1_) including wound class and operation time; this improved both the C-statistic, to 0.702 (95% CI 0.682, 0.723) and calibration O/E ratio to 1.000 (IQR 0.990, 1.023). Thirdly, a revised M_1_ model (called M_2_) was updated by fitting four additional significant predictive factors derived from the data (i.e. blood transfusion, concurrent procedure, diabetes mellitus, emergency status) further improving the C-statistic to 0.768 (95% CI 0.746, 0.790) with O/E ratio of 1.000 (IQR 0.996,1.003). Fourthly, an M_3_ model re-estimated the coefficient for all four original predictive factors based on the data; only three of the four factors remained significant (i.e. operation time > 2 h, contaminated/dirty-infected wounds and the number of diagnoses ≥ 3). However, this model performed less well in comparison with M_2_ with a C-statistic of 0.702 (95% CI 0.682, 0.723) and an O/E ratio of 1.000 (IQR 0.990, 1.023). Fifthly, a revised SENIC (called M_4_) was updated by incorporating the M_2_ and M_3_ models with the three original significant predictors (operation time >2 h, contaminated/dirty-infected wounds, ≥3 diagnoses) plus four additional significant predictors derived from the data (blood transfusion, concurrent procedure, diabetes mellitus, emergency status). All except contaminated/dirty-infected wounds and the number of diagnoses ≥3 remained significant and were retained in the final updated model providing a C-statistic of 0.764 (95% CI 0.742, 0.787) and O/E ratio of 1.000 (IQR 0.998, 1.037).

**Table 2.** External validation of the study on the efficacy of nosocomial infection control (SENIC) model.

Model	Equation			Goodness of fit			O/E ratio		
	Equation^a^	Factor, *n*	C-statistic (95% CI)	H-L	df	*P*-value	P25	P50	P75
M_0_	lnP1−P=b0+b1 score	4	0.668 (0.648, 0.688)	21.60	1	*P* < 0.05	0.750	0.998	1.047
M_1_	$ln\left[\frac{P}{1-P}\right]=-2.06$ $+0.75xM_{0}$ +1.01xORTime −0.84xWoundClass	4	0.702 (0.682, 0.723)	0.26	1	*P* = 0.613	0.990	1.000	1.023
M_2_	$ln\left[\frac{P}{1-P}\right]=-4.89$ +23.09*xM*_1_ +0.99xBloodTx +0.51xConProc +0.54xDM +0.30xEmergency	8	0.768 (0.746, 0.790)	6.19	3	*P* = 0.103	0.996	1.000	1.003
M_3_	$ln\left[\frac{P}{1-P}\right]=-4.55$ +1.78xORTime +0.07*xWoundClass*−0.64*xNdiagnosis*	3	0.702 (0.682, 0.723)	0.26	1	*P* = 0.613	0.990	1.000	1.023
M_4_	$ln\left[\frac{P}{1-P}\right]=-4.81$ +1.35xORTime +0.97xBloodTx +0.53xConProc +0.51xDM +0.31xEmergency	5	0.764 (0.742, 0.787)	1.38	3	*P* = 0.710	0.998	1.000	1.037
M_5_	$ln\left[\frac{P}{1-P}\right]=-5.19$ + 0.94xBloodTx + 0.65xConProc +0.53xDM +0.32xEmergency +0.37xOpenSurg +1.39xORTime	6	0.768 (0.745, 0.790)	1.84	3	*P* = 0.606	0.976	0.999	1.004

a Original SENIC Score = –4.48 + 1.12xAbdominal + 1.04xORTime + 1.04xWoundClass + 0.86xNdiagnosis; where Abdominal = Abdominal surgery, BloodTx = Blood transfusion, ConProc = Concurrent procedure ≥ 2, DM = Diabetes mellitus, Ndiagnosis = The number of diagnoses ≥ 3, Emergency = Emergency, OpenSurg = Open surgery, ORTime = Operation time > 2 h, WoundClass = Wound class (contaminated or dirty).

O/E, observed/expected; CI, confidence interval; H-L, Hosmer-Lemeshow; df, degree of freedom; P25, 25^th^ percentile; P50, 50^th^ percentile; P75, 75^th^ percentile.

Finally, any of the predictors removed in the previous steps were re-considered within the M_5_ model (i.e. updated-SENIC-LR model), which indicated that the inclusion of open-surgery could further improve the C-statistic to 0.768 (95% CI 0.745, 0.790) and O/E ratio of 0.999 (IQR 0.976, 1.004). The equation derived for the M_5_ model was as follows:

$$ln\left[\frac{P}{1-P}\right]=-5.192\;+1.391x\mathrm{ORTime}+0.939x\mathrm{BloodTx}\;+0.650x\mathrm{ConProc}+0.529xDM+0.316x\mathrm{Emergency}+0.369x\mathrm{OpenSurg}\;$$

where ORTime = operation time > 2 versus  ≤ 2 h, BloodTx = blood transfusion versus no-blood transfusion, ConProc =concurrent procedure < 2 versus concurrent procedure >2, DM = diabetes mellitus versus non-diabetes mellitus, Emergency =emergency versus non-emergency, and OpenSurg = open versus non-open surgery.

Four ML approaches were applied considering the 14 factors listed in Table 1 as follows: firstly, a decision tree was constructed following the model hyperparameters (see supplementary materials, Table S3). The C-statistic and recall for grid searching were 0.759 (95% CI 0.741, 0.778) and 0.884 (95% CI 0.847, 0.919) in the training dataset, respectively, which were reduced to 0.627 (95% CI 0.579, 0.671) and 0.613 (95% CI 0.520, 0.701) in the testing dataset, respectively (Table 3). Manual searching improved overfitting, i.e. reduced the difference in performance between the training and testing datasets with corresponding metrics of 0.753 (95% CI 0.731, 0.776) and 0.805 (95% CI 0.762, 0.848) in the training dataset respectively; and 0.651 (95% CI 0.604, 0.698) and 0.594 (95% CI 0.500, 0.687) in the testing dataset.

**Table 3.** Machine learning performance characteristics for abdominal surgical site infection classification.

	Grid search		Manual search	
	Training *n* = 8817	Test *n* = 3779	Training *n* = 8817	Test *n* = 3779
Decision tree				
Accuracy (95% CI)	0.644 (0.633, 0.654)	0.639 (0.623, 0.654)	0.705 (0.695, 0.715)	0.705 (0.691, 0.720)
Recall (95% CI)	0.884 (0.847, 0.919)	0.613 (0.520, 0.701)	0.805 (0.762, 0.848)	0.594 (0.500, 0.687)
Specificity (95% CI)	0.635 (0.624, 0.645)	0.639 (0.624, 0.655)	0.701 (0.692, 0.712)	0.709 (0.694, 0.723)
Precision (95% CI)	0.079 (0.070, 0.088)	0.045 (0.034, 0.056)	0.088 (0.077, 0.098)	0.053 (0.040, 0.067)
C-statistic (95% CI)	0.759 (0.741, 0.778)	0.627 (0.579, 0.671)	0.753 (0.731, 0.776)	0.651 (0.604, 0.698)
F1 Score (95% CI)	0.146 (0.130, 0.161)	0.083 (0.065, 0.103)	0.158 (0.141, 0.175)	0.097 (0.075, 0.121)
Random forest				
Accuracy (95% CI)	0.971 (0.967, 0.974)	0.944 (0.937, 0.952)	0.679 (0.669, 0.690)	0.676 (0.662, 0.692)
Recall (95% CI)	0.917 (0.885, 0.946)	0.129 (0.067, 0.198)	0.838 (0.797, 0.880)	0.633 (0.538, 0.723)
Specificity (95% CI)	0.972 (0.969, 0.976)	0.967 (0.961, 0.973)	0.673 (0.664, 0.684)	0.677 (0.663, 0.693)
Precision (95% CI)	0.546 (0.503, 0.588)	0.097 (0.050, 0.152)	0.083 (0.074, 0.093)	0.051 (0.039, 0.064)
C-statistic (95% CI)	0.945 (0.929, 0.960)	0.548 (0.517, 0.583)	0.755 (0.735, 0.778)	0.656 (0.607, 0.702)
F1 Score (95% CI)	0.684 (0.646, 0.720)	0.110 (0.058, 0.169)	0.152 (0.136, 0.169)	0.095 (0.074, 0.118)
XGBoost				
Accuracy (95% CI)	0.958 (0.954, 0.963)	0.908 (0.899, 0.919)	0.712 (0.704, 0.722)	0.713 (0.700, 0.727)
Recall (95% CI)	1	0.158 (0.093, 0.233)	0.855 (0.817, 0.894)	0.633 (0.543, 0.724)
Specificity (95% CI)	0.957 (0.953, 0.962)	0.929 (0.921, 0.938)	0.708 (0.699, 0.718)	0.716 (0.701, 0.730)
Precision (95% CI)	0.455 (0.417, 0.494)	0.058 (0.033, 0.087)	0.094 (0.084, 0.105)	0.057 (0.044, 0.072)
C-statistic (95% CI)	0.979 (0.977, 0.981)	0.544 (0.511, 0.582)	0.781 (0.762, 0.802)	0.675 (0.628, 0.720)
F1 Score (95% CI)	0.626 (0.588, 0.661)	0.085 (0.049, 0.125)	0.170 (0.152, 0.187)	0.106 (0.082, 0.130)
Naïve Bayes				
Accuracy (95% CI)	0.712 (0.704, 0.722)	0.713 (0.699, 0.727)	0.648 (0.639, 0.659)	0.655 (0.641, 0.671)
Recall (95% CI)	0.640 (0.586, 0.695)	0.574 (0.474, 0.661)	0.776 (0.728, 0.822)	0.703 (0.614, 0.784)
Specificity (95% CI)	0.715 (0.706, 0.725)	0.716 (0.703, 0.731)	0.643 (0.634, 0.654)	0.654 (0.640, 0.700)
Precision (95% CI)	0.074 (0.064, 0.084)	0.053 (0.039, 0.066)	0.071 (0.063, 0.081)	0.053 (0.042, 0.065)
C-statistic (95% CI)	0.678 (0.650, 0.704)	0.645 (0.594, 0.690)	0.710 (0.686, 0.733)	0.679 (0.632, 0.722)
F1 Score (95% CI)	0.133 (0.116, 0.150)	0.097 (0.073, 0.120)	0.131 (0.116, 0.146)	0.100 (0.078, 0.120)

CI, confidence interval.

Random forest was constructed according to the model hyperparameters (see supplementary materials, Table S3). For grid searching, performance was very high in the training dataset (i.e. C-statistic and recall were 0.945 (95% CI 0.929, 0.960) and 0.917 (95% CI 0.885, 0.946), respectively, but these values were significantly reduced for the testing dataset reflecting an unreliable model with corresponding metrics of 0.548 (95% CI 0.517, 0.583) and 0.129 (95% CI 0.067, 0.198) (Table 3). Applying a manual search performed less well in the training dataset than grid searching, with a C-statistic and recall values of 0.755 (95% CI 0.735, 0.778) and 0.838 (95% CI 0.797, 0.880), respectively, but improved model overfitting for the testing dataset with corresponding values of 0.656 (95% CI 0.607, 0.702) and 0.633 (95% CI 0.538, 0.723), respectively.

The XGBoost model was constructed according to the model hyperparameters (see supplementary materials, Table S3) with the performance shown in Table 3. The grid searching C-statistic and recall for the training dataset were 0.979 (95% CI 0.977, 0.981) and 1, respectively, with significantly lower corresponding values in the testing dataset of 0.544 (95% CI 0.511, 0.582) and 0.158 (95% CI 0.093, 0.233). Likewise, manual searching improved performance in the training dataset compared with the testing dataset with values of 0.781 (95% CI 0.762, 0.802) and 0.855 (95% CI 0.817, 0.894) for the former, respectively; and 0.675 (95% CI 0.628, 0.720) and 0.633 (95% CI 0.543, 0.724), respectively, for the testing dataset.

The Naïve Bayes model was constructed according to the model hyperparameters (see supplementary materials, Table S3). Model performance did not differ significantly between training and testing datasets (Table 3). C-statistic and recall were 0.678 (95% CI 0.650, 0.704) and 0.640 (95% CI 0.586, 0.695) in the training dataset, respectively, with corresponding values of 0.645 (95% CI 0.594, 0.690) and 0.574 (95% CI 0.474, 0.661), respectively, in the testing dataset. Likewise the manual searching values were 0.710 (95% CI 0.686, 0.733) and 0.776 (95% CI 0.728, 0.822), respectively, in the training dataset; and 0.679 (95% CI 0.632, 0.722) and 0.703 (95% CI 0.614, 0.784), respectively, in the testing dataset.

The assessment of factor contributions was performed by estimating SHAP values (see supplementary materials, Figure S2). For the decision tree model, the five most important factors were operation time, concurrent procedure, emergency status, age and pre-operative antibiotics use, in which operation time, concurrent procedure and pre-operative antibiotics use showed lowering effects, whereas emergency status and age had unclear contributions in both preventive and risk effects. The random forest suggested the largest contribution was from operation time, followed by concurrent procedure, blood transfusion, age and pre-operative antibiotics use, all of which were negatively associated with SSI (see supplementary materials, Figure S3). The XGBoost model identified operation time as the largest contributory factor, followed by age, concurrent procedure, blood transfusion and pre-operative antibiotics use (see supplementary materials, Figure S4). All factors were associated with increased SSI risk, except age where the direction of effect was unclear.

The Naïve Bayes model indicated that operation time, concurrent procedure, blood transfusion, emergency status and ICU were the five most important factors that contributed to SSI risk (see supplementary materials, Figure S5). The direction of effect for the four predictive factors were similar to the updated-SENIC-LR model, except for open surgery, which was positively associated with a preventative effect in the Naïve Bayes model. In addition, a SHAP dependence plot was constructed to explain how individual contributions of operation time and four other predictors (emergency status, blood transfusions, ICU admission and number of concurrent procedures) influenced SSI risk. This plot revealed that patients with operation times >2 h and those undergoing concurrent procedures had the strongest contributions to SSI risk, followed by emergency status, blood transfusions and ICU admission (see supplementary materials, Figure S6). To further explore individual predictions, the data were divided into three groups based on tertile distributions of SSI probability: low, intermediate and high risk. A SHAP force plot was constructed to visualize these predictions, which also indicated a high risk of SSI in patients who underwent operations >2 h and had concurrent procedures (see supplementary materials, Figure S7).

## Discussion

This current study performed external validation of the original SENIC model based on four factors including abdominal surgery, operation time, wound class and the number of diagnosed conditions diagnosed, which were all variables significantly associated with SSI in the data. The original model showed fair performance with a C-statistic of 0.668. Five additional factors that were significantly associated with SSI that improved SSI classification, including blood transfusion, concurrent procedure, diabetes mellitus, emergency status and open surgery, were evaluated with the final model showing an improved C-statistic of 0.768. In addition, four ML approaches including decision tree, random forest, XGBoost and Naïve Bayes were used to develop SSI prediction models using 14 predictive factors. The Naïve Bayes approach provided the best discrimination, followed by XGBoost, random forest and decision tree with C-statistics of 0.679, 0.675, 0.656 and 0.651, respectively. However, all ML models performed sub-optimally in comparison with the updated-SENIC-LR model.

Machine learning parameters were refined by grid search and manual tuning, with the former leading to greater overfitting, i.e. the C-statistics between the training and testing datasets differed significantly from 4.8%–44.4% and 4.3%–13.6%, respectively. The Naïve Bayes approach was subject to the least overfitting, followed by random forest, decision tree and XGBoost with relative differences of 4.3%, 13.1%, 13.5% and 13.6%, respectively. This may be due to the Naïve Bayes algorithm classifying samples on the basis of probability thresholds. The random forest addresses the issue of overfitting by generating many decision trees, but this provided limited improvement. XGBoost provides a sequence classifier by incorporating multiple trees with regularization parameters but this produced similar findings to the random forest and showed the highest level of overfitting.

These current findings identified two clinically useful predictive models associated with SSI risk as measured by discrimination and calibration performance. First, the updated-SENIC-LR required six predictive factors routinely collected during surgery (i.e. operation time, blood transfusion, concurrent procedure, emergency status, diabetes mellitus and open surgery). Secondly, the Naïve Bayes approach required all 14 factors including two demographic variables (age, sex), five pre-surgical variables (diabetes mellitus, number of diagnoses, emergency status, ASA, pre-operative antibiotics use) and seven peri-/post-surgical data (concurrent procedures, anaesthesia type, wound class, operation time, blood transfusion, surgical approach, admission ward after surgery). The updated-SENIC-LR and Naïve Bayes models have now been installed on our hospital server to provide access for clinicians (http://www3.ra.mahidol.ac.th/ramaml). Use by clinical staff for further prospective performance evaluation prior to recommendations for wider routine clinical practice will be encouraged. Error analysis (i.e. false-positive and false-negative classifications) will also be explored to improve model performance.

This current study had several strengths. First, systematic model revision and updating provided significant improvement in model performance. Operation time and wound class were identified as key factors in 19 of the 33 studies in the review (see supplementary materials, Figure S1), with operation time being particularly significant and aligning with the SENIC model. Additionally, operation time contributed the most among the top five factors in the Naïve Bayes model, followed by concurrent procedures, blood transfusion, emergency and ICU admission, all of which have been used in previous studies. Secondly, ML approaches were developed using different algorithms to identify the optimum approach for SSI prediction.

However, this current study had several limitations. The models were developed using a cohort of high-risk patients undergoing gastrointestinal surgery with contaminated wounds and hernia surgery with clean-contaminated wounds involving foreign bodies. While the models may be applicable to these specific types of surgeries, their performance on other abdominal procedures, such as hepatobiliary and pancreatic surgery, may be less accurate. Overfitting was observed during ML model development, potentially due to the relatively small number of SSIs compared with the number of predictive factors used in model training. Although a balanced class weight was employed to mitigate bias towards the majority class, overfitting persisted. Up-sampling was not implemented due to the risk of further overfitting, while down-sampling could have led to information loss. In addition, some predictive factors were unexplainable as per the SHAP analysis findings, making their explainability less transparent. For instance, operation time >2 h, blood transfusion, concurrent procedure, open surgery and diabetes mellitus should increase SSI risks but several ML approaches failed to consistently support the direction and magnitude of these effects.

In conclusion, the updated-SENIC-LR and Naïve Bayes models may offer clinical utility in SSI prediction following abdominal surgery. However, both models require further prospective evaluation in other clinical datasets before deployment in a clinical setting given their predictive ability is still modest.

## Supplemental Material

sj-pdf-1-imr-10.1177_03000605241293696 - Supplemental material for Can machine learning models improve the prediction of surgical site infection in abdominal surgery than traditional statistical models? Supplemental material, sj-pdf-1-imr-10.1177_03000605241293696 for Can machine learning models improve the prediction of surgical site infection in abdominal surgery than traditional statistical models? by Pongsathorn Piebpien, Amarit Tansawet, Oraluck Pattanaprateep, Anuchate Pattanateepapon, Chumpon Wilasrusmee, Gareth J. Mckay, John Attia and Ammarin Thakkinstian in Journal of International Medical Research

## Data statement

Data are available from the corresponding author upon reasonable request. The codes available at https://www.rama.mahidol.ac.th/ceb/codes/code_pj2

## Trial registration

This study was retrospectively registered at the Thai Clinical Trials Registry on 17/10/2022 and approved on 18/10/2022 (no. TCTR20221018001).
