# Supplementary material for: Can machine learning models improve the prediction of surgical site infection in abdominal surgery than traditional statistical models?
Source: J Int Med Res. 2024 Nov 17;52(11):03000605241293696. doi: 10.1177/03000605241293696 (PMC11571240; doi:10.1177/03000605241293696)
Supplement: sj-pdf-1-imr-10.1177_03000605241293696 - Supplemental material for Can machine learning models improve the prediction of surgical site infection in abdominal surgery than traditional statistical models? [file sj-pdf-1-imr-10.1177_03000605241293696.pdf]

Can machine learning models improve the prediction of surgical site infection in abdominal surgery than traditional statistical models?

Supporting information

Table S1. The ICD-9-CM procedure classification

Table S2. Factors descriptions

Table S3. Hyperparameter descriptions for machine learning models

Figure S1. Systematic review article screening protocol

Figure S2. Decision tree model with SHapley Additive exPlanations (SHAP) value impact

Figure S3. Random forest model with SHapley Additive exPlanations (SHAP) value impact

Figure S4. XGBoost model with SHapley Additive exPlanations (SHAP) value impact

Figure S5. Naïve Bayes model with SHapley Additive exPlanations (SHAP) value impact

Figure S6. SHAP dependence contribution plot of operation time and other important features suggested in the Naïve Bayes Model

Figure S7. A SHAP force plot for risk classification: A Naïve Bayes model

Table S1. The ICD-9-CM procedure classification

Code	Classification
43	Incision and excision of the stomach
44	Other operations on the stomach
45	Incision, excision, and anastomosis of the intestine
46	Other operations on the intestine
48	Operations on the rectum, rectosigmoid and perirectal tissue
49	Operations on the anus
53	Repair of hernia

Table S2. Factors descriptions

Factors	Description	Range/Values
Sex	Sex, consisted of male and female	1= male, 0=female
Age	Age at operation (year)	18-104
ASA class	American Society of Anesthesiologists classification, consisted of class 1 to class 5	1, 2, 3, 4, 5
Diabetes mellitus	History of diabetes mellitus	1= yes, 0=no
No. of diagnosis	Number of comorbidity diagnosis	1=≥3, 0=<3
Emergency	Emergency status, consisted of elective and emergency	1= Emergency, 0= elective
Pre-operative antibiotics	Use and not-use antibiotics before surgery	1=yes, 0= no
Surgical approach	Surgical approaches, consisted of open and laparoscopic/robotic surgeries	1=open surgery, 0=non-open surgery
Concurrent procedure	Number of concurrent procedure	1=≥2, 0=<2
OR time	Operation time since incision to finish operation (hour)	1=>2, 0=≤2
Wound class	Wound class, consisted of contaminated/dirty and cleaned/cleaned-contaminated wounds	1=contaminated/dirty wound, 0= clean/clean-contaminated wound
Epidural anesthesia	Type of anesthesia, consisted of general and local anesthesia	1=general anesthesia, 0=local anesthesia

Factors	Description	Range/Values
ICU	Admitted at ICU	1=yes, 0=no
Blood transfusion	received blood transfusion	1=yes, 0=no

Table S3. Hyperparameter descriptions for machine learning models

Model	Hyperparameter	Grid search	Manual search
**Decision tree**	Class weight	balanced	balanced
	Criterion	gini	gini
	Max depth	7	5
	Max features	6	7
	Min sample leaf	3	4
	Min sample split	9	8
**Random forest**	N estimators	500	400
	Class weight	balanced	balanced
	Criterion	gini	gini
	Max depth	25	5
	Max features	5	10
	Min sample leaf	2	1
	Min sample split	2	15
**XGBoost**	Learning rate	0.55	0.04
	N estimators	1000	300
	scale_pos_weight	pos_ratio	pos_ratio
	Max depth	4	3
	gamma	0	0
	lamda	1	1
	alpha	0	0
**Naïve Bayes**	Variable smoothing	1.912556e-09	0.289999e-09

Figure S1. Systematic review article screening protocol

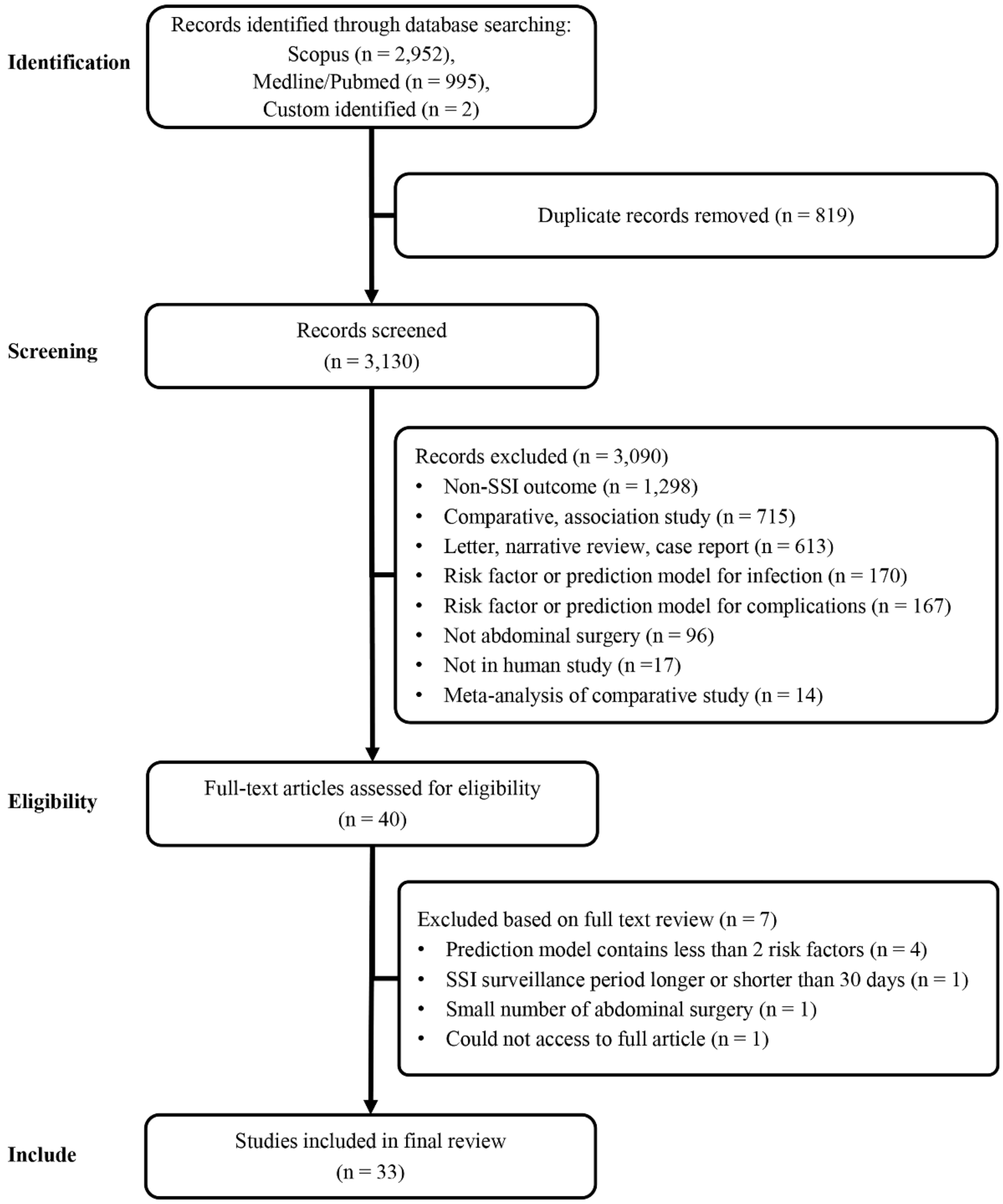

Figure S2. Decision tree model with SHapley Additive exPlanations (SHAP) value impact

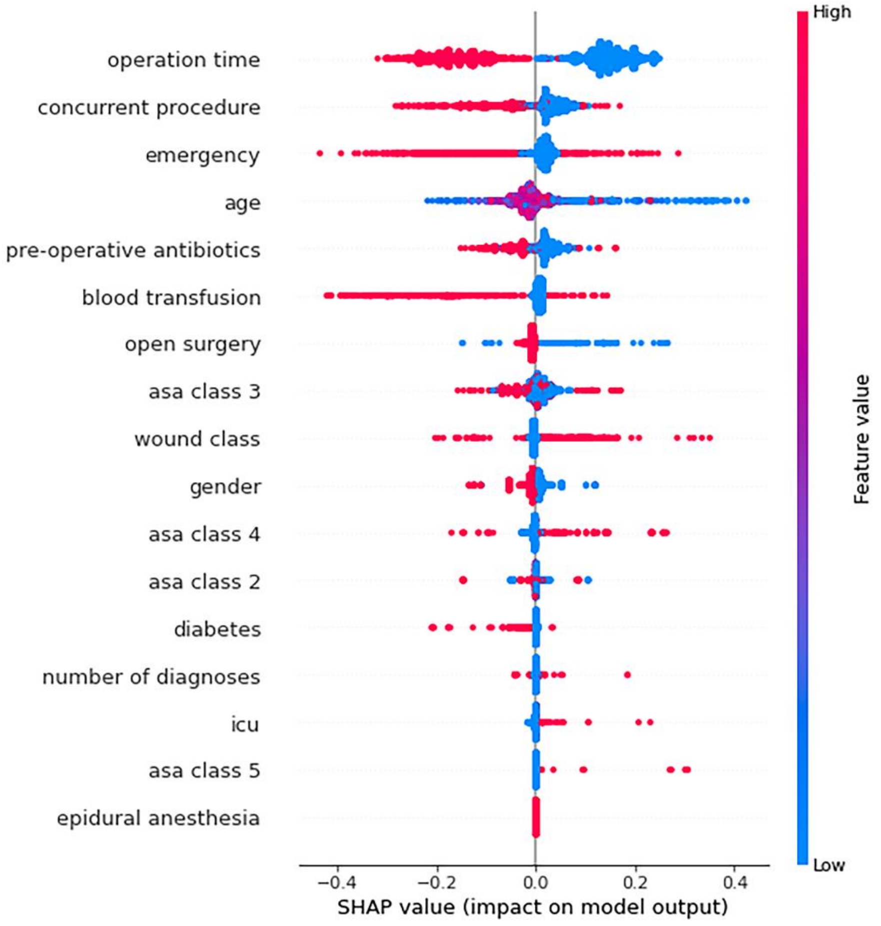

Figure S3. Random forest model with SHapley Additive exPlanations (SHAP) value impact

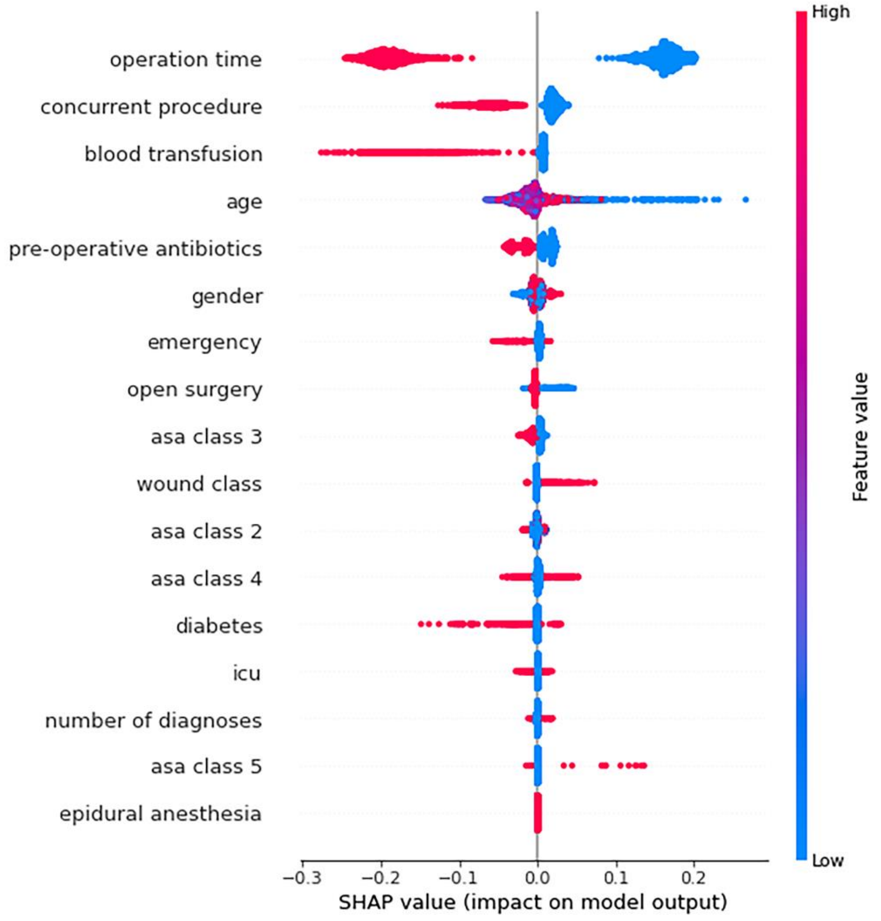

Figure S4. XGBoost model with SHapley Additive exPlanations (SHAP) value impact

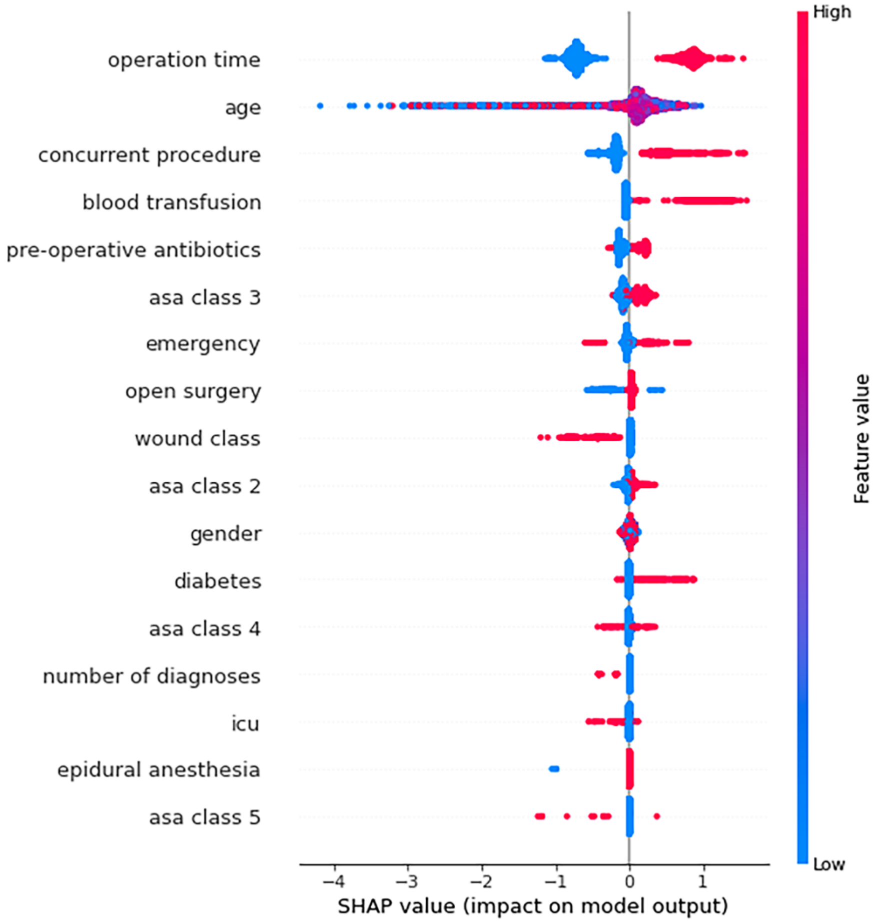

Figure S5. Naïve Bayes model with SHapley Additive exPlanations (SHAP) value impact

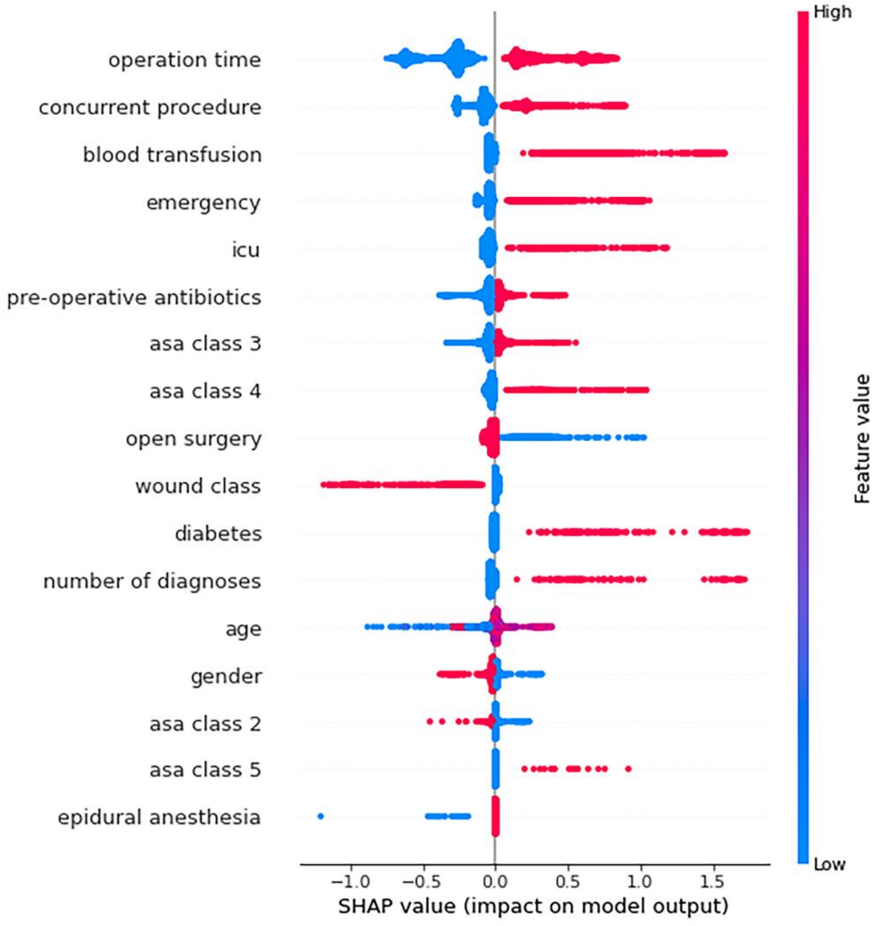

Figure S6. SHAP dependence contribution plot of operation time and other important features suggested in the Naïve Bayes Model

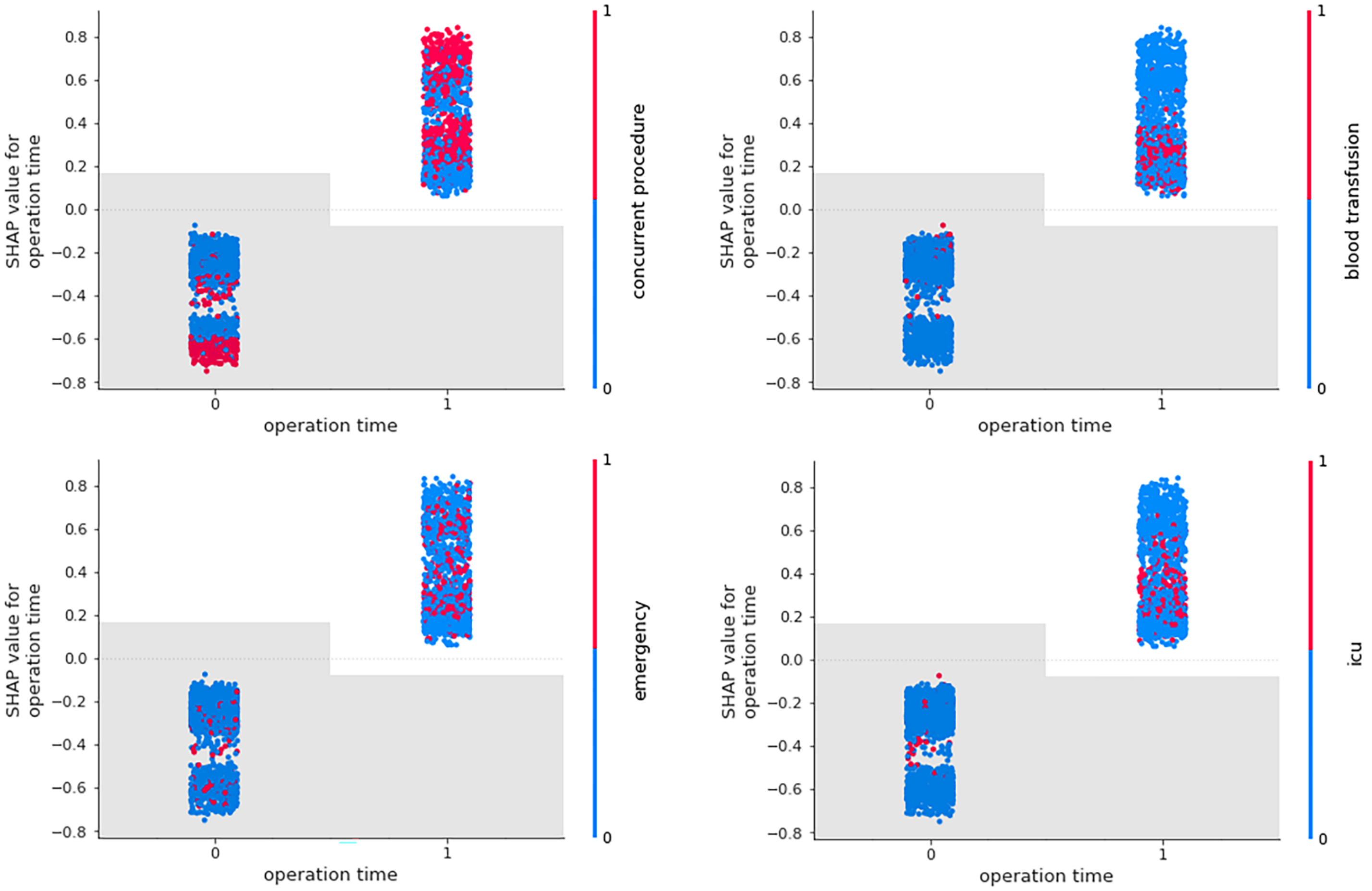

Figure S7. A SHAP force plot for risk classification: A Naïve Bayes model

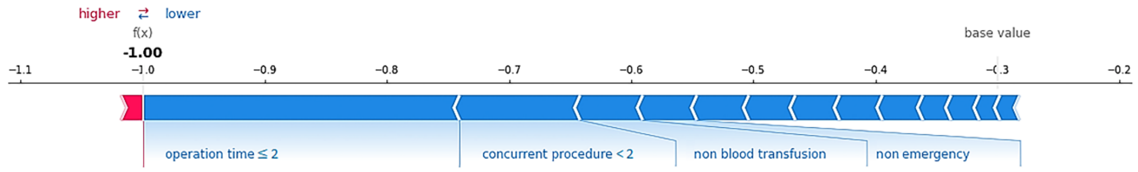

(a) Low risk (first tertile)

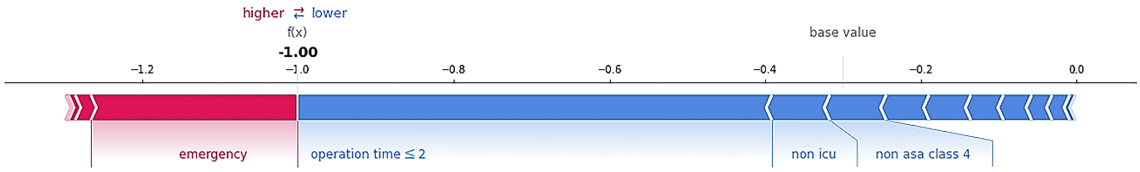

(b) Intermediate risk (second tertile)

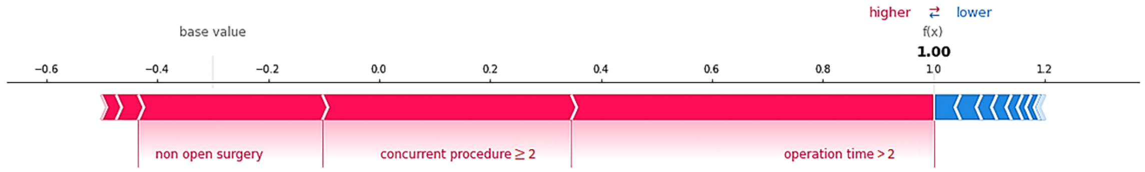

(c) High risk (third tertile)
